# Author Correction: Lkb1 suppresses amino acid-driven gluconeogenesis in the liver

**DOI:** 10.1038/s41467-021-22104-4

**Published:** 2021-03-17

**Authors:** Pierre-Alexandre Just, Sara Charawi, Raphaël G. P. Denis, Mathilde Savall, Massiré Traore, Marc Foretz, Sultan Bastu, Salimata Magassa, Nadia Senni, Pierre Sohier, Maud Wursmer, Mireille Vasseur-Cognet, Alain Schmitt, Morgane Le Gall, Marjorie Leduc, François Guillonneau, Jean-Pascal De Bandt, Patrick Mayeux, Béatrice Romagnolo, Serge Luquet, Pascale Bossard, Christine Perret

**Affiliations:** 1 Université de Paris, Institut Cochin, INSERM, CNRS, F75014 Paris, France; 2 APHP, Centre–Université de Paris, Paris, France; 3 Unité de Biologie Fonctionnelle et Adaptative, Centre National la Recherche Scientifique, Unité Mixte de Recherche 8251, Université Paris Diderot, Sorbonne Paris Cité, 75205 Paris, France; 4 EA4466, PRETRAM, Université Paris Descartes, Paris, France; 5 UMR IRD 242, UPEC, CNRS 7618, UPMC 113, INRA 1392, Sorbonne Universités Paris and Institut d’Ecologie et des Sciences de l’Environnement de Paris, Bondy, France; 6 Electron Miscroscopy Facility, Institut Cochin, F75014 Paris, France; 7 3P5 proteom’IC Facility, Université de Paris, Institut Cochin, INSERM, CNRS, F-75014 Paris, France

**Keywords:** Physiology, Metabolism

Correction to: *Nature Communications* 10.1038/s41467-020-19490-6, published online 30 November 2020.

This Article contained an error in the Data Availability statement. Reference to the phosphoproteome data of refed animals, deposited to the PRIDE repository, was accidentally omitted. The data are accessible via accession number PXD019755. This information has now been added to the Data Availability statement for both the PDF and HTML versions of this Article, as well as the Reporting Summary.

