# Supplementary material for: Lkb1 suppresses amino acid-driven gluconeogenesis in the liver
Source: Nat Commun. 2020 Nov 30;11:6127. doi: 10.1038/s41467-020-19490-6 (PMC7705018; doi:10.1038/s41467-020-19490-6)
Supplement: Supplementary file 1 — Supplementary Information [file 41467_2020_19490_MOESM1_ESM.pdf]

Supplementary Information

Lkb1 suppresses amino acid-driven gluconeogenesis in the liver

Just PA, Charawi S, et al.

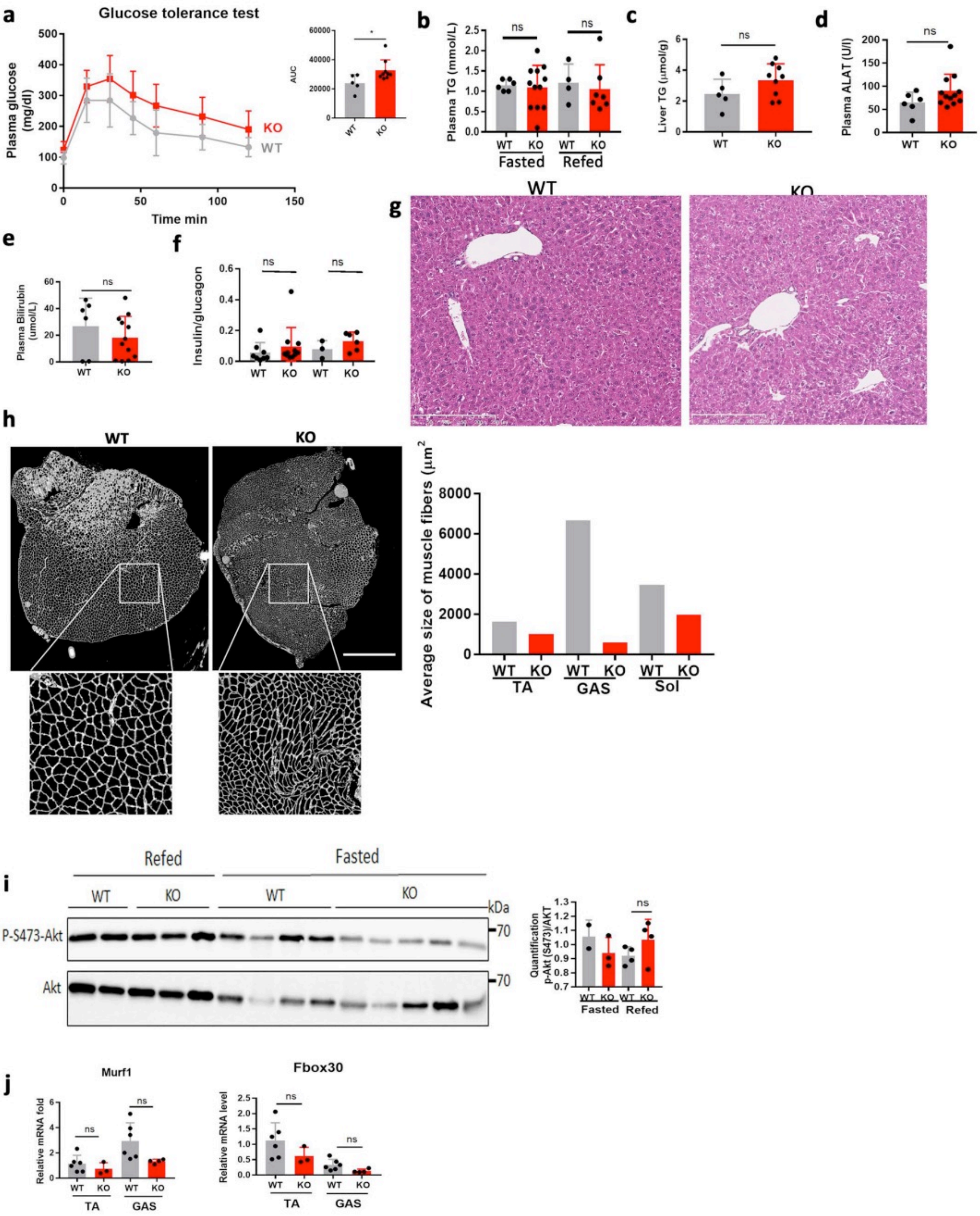

Supplementary Figure 1. Phenotypes of Lkb1KO^{livad}mice.

a: Glucose tolerance test in mutant Lkb1KO^{livad} (KO, n=9) and control (WT, n=5) mice. The area under the curve (AUC) of glucose level in the GTT is shown. Data are means ± SD. P values were determined by unpaired two-tailed t-test. *p ≤ 0.05. b: Plasma triglyceride concentration in fasted mutant Lkb1KO^{livad} (KO, n=12) and control (WT, n=6) mice and refed mutant Lkb1KO^{livad} (KO, n=7) and control (WT, n=4) mice. Data are means ± SD. P values were determined by unpaired two-tailed t-test. ns: not significant. c: Liver triglyceride content in fasted mutant Lkb1KO^{livad} (KO, n=9) and control (WT, n=5) mice. Data are means ± SD. P values were determined by unpaired two-tailed t-test. ns: not significant. d: Plasma ALT and

bilirubin levels in fasted mutant Lkb1KO^{livad} (KO, n=12) and control (WT, n=6) mice. Data are means ± SD. P values were determined by unpaired two-tailed t-test. ns: not significant. e: Plasma bilirubin levels in fasted mutant Lkb1KO^{livad} (KO, n=12) and control (WT, n=6) mice. Data are means ± SD. P values were determined by unpaired two-tailed t-test. ns: not significant. f: Plasma insulin to glucagon ratio of fasted Lkb1KO^{livad} (KO, n=11) and control (WT, n=8) mice and refed Lkb1KO^{livad} (KO, n=6) and control (WT, n=3) mice. Data are means ± SD. P values were determined by unpaired two-tailed t-test. ns: not significant. g: H&E of WT and Lkb1KO^{livad}mice. Representative image of three independent experiments. h: Skeletal muscle fiber size in mutant (KO, n=1) and control (WT, n=1) mice. It was analyzed by dystrophin immunohistochemistry showing all the fibers were smaller in mutant compared to control mice. The quantification of fiber size, and shown on the right, was done with metamorph software on microscopic acquisitions done at x10 magnification. TA: tibialis, GAS: gastrocnemius, Sol: Soleus. i: Western blot analysis of the Akt signaling in skeletal muscle of fasted (KO, n=4; WT, n=5) and refed (KO, n=3; WT, n=2) mutant and control mice. Quantification was performed with FUJI multigauge software. Data are means ± SD. P values were determined by unpaired two-tailed t-test. ns: not significant. j: RT-qPCR analysis of the expression of ubiquitin ligase of the skeletal muscle *Murf1* and *Fbox32* of mutant (n=4) and WT (n=6) mice. Data are means ± SD. P values were determined by unpaired two-tailed t-test. ns: not significant.

Source data are provided as a Source Data file.

For the experiments described in a, b, c, d, e, f, g, h, i, animals were analyzed 15 days after the injection of tamoxifen.

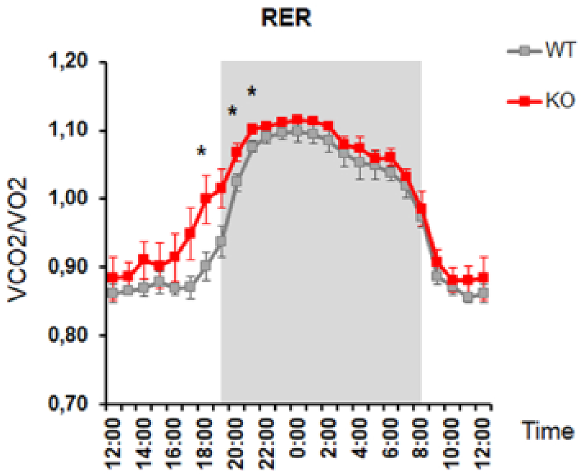

Supplementary Figure 2. Respiratory exchange ratio.
Daylight, night and 24 h mean respiratory exchange ratio (VCO_2/VO_2) of mutant Lkb1KO^{livad} (KO, n= 7) and control (WT= 6) mice. Data are expressed as the mean ± SEM of 6 animals per group. P values were determined by Wilcoxon–Mann–Whitney tests. *$p \leq 0.05$.

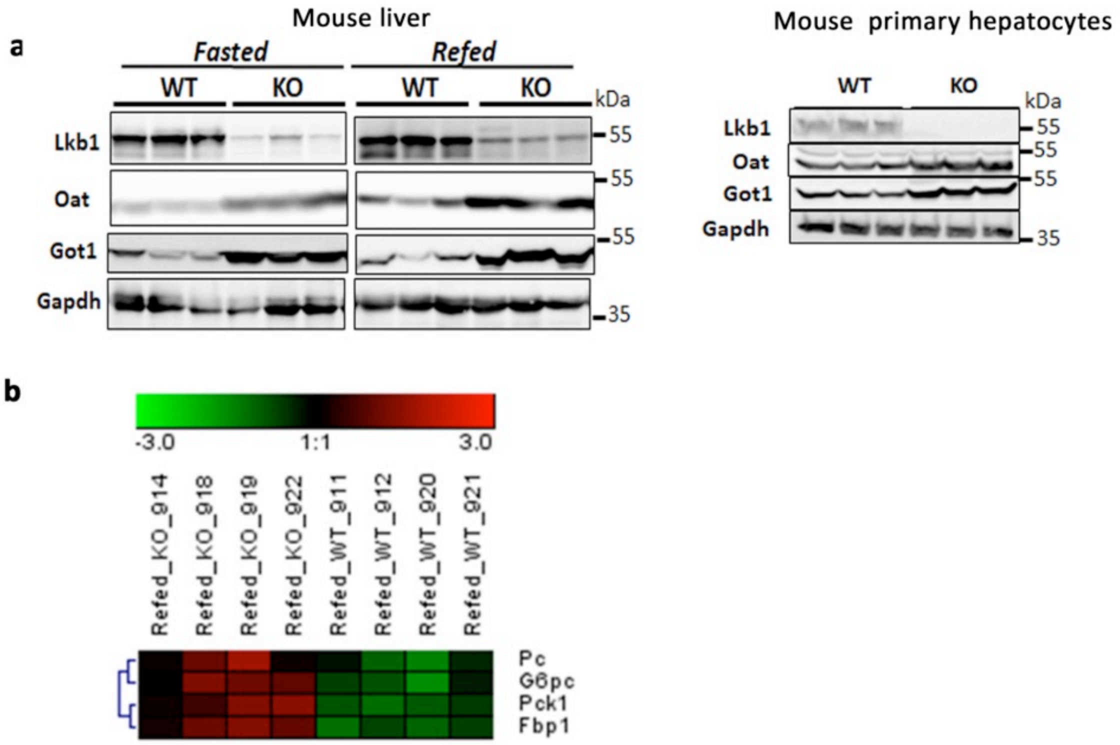

Supplementary Figure 3. Increase amino acid catabolism and gluconeogenesis in Lkb1KO^{livad}mice.

a: Immunoblot analysis of Oat and Got1 in mouse liver and primary hepatocytes from Lkb1KO^{livad} (KO, n=3) mice and controls (WT, n=3). Representative blot of two independent experiments. b: Heatmap of gluconeogenic proteins identified by proteomic analysis in refed KO and WT animals

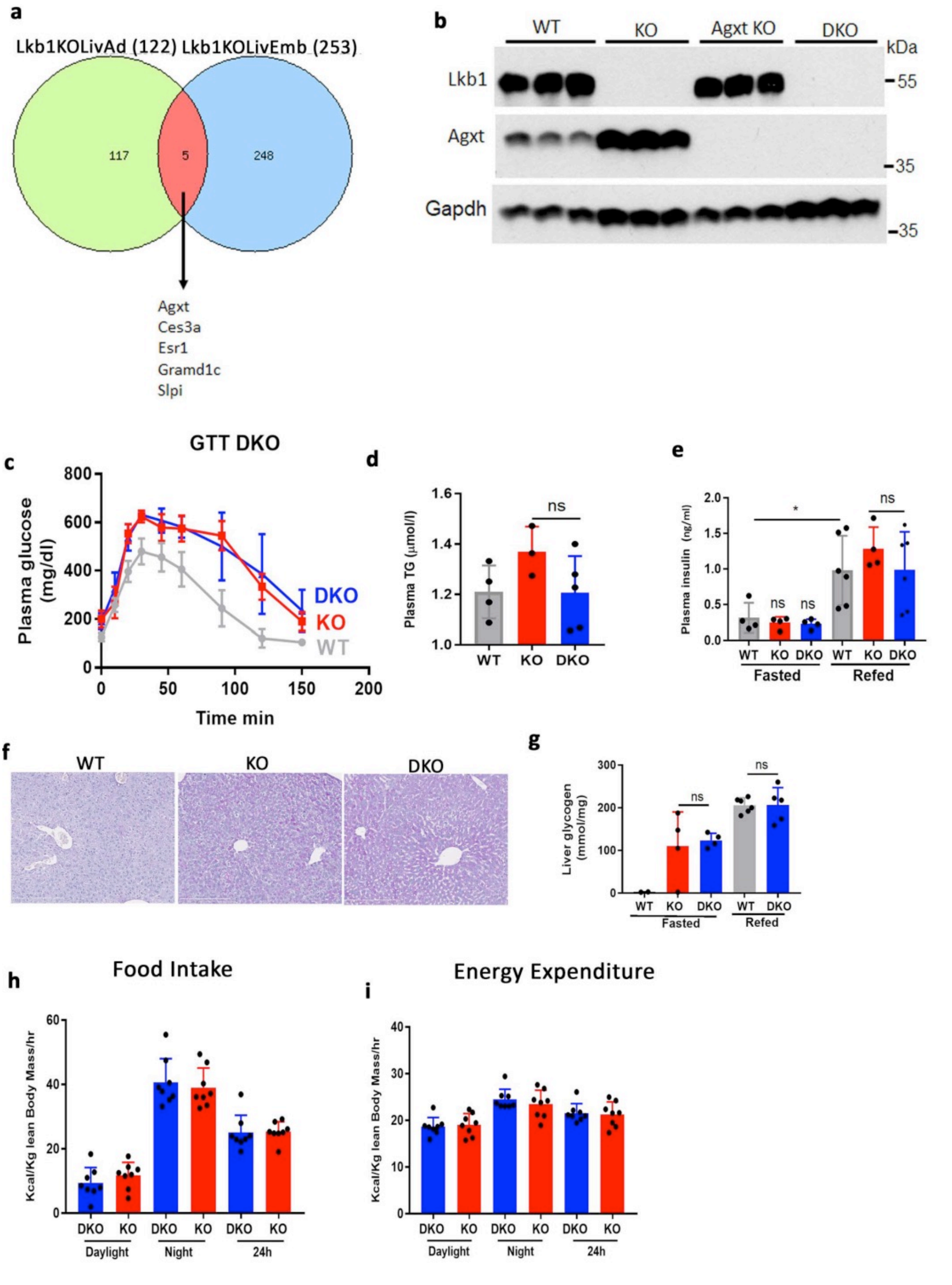

Supplementary Figure 4. Phenotypes of DKO (Lkb1KO^{livad};AgxtKO) mice.

a: Venn diagram of genes expressed specifically in mice with either a liver-specific deletion of *Lkb1* in adult hepatocytes (Lkb1KO^{livad}) or a liver-specific deletion of *Lkb1* specifically in the embryonic liver (Lkb1KO^{livEmb}) [1]. These results were obtained by microarray analysis. b: Immunoblot analysis of Lkb1 and Agxt in WT, Lkb1KO^{livad} (KO, n=3) mice; Agxt KO (n=3)

mice and in mice lacking both *Lkb1* and *Agxt* (DKO, n=3). c: Glucose tolerance test in DKO (DKO, n=4), Lkb1KO^{livad} (KO, n=9) and control (WT, n=5) mice. d: Plasma TG in DKO (n=5), K(n=3) and WT (n=5) in fasted mice. Data are means ± SD. P values were determined by unpaired two-tailed t-test. ns: not significant. e: Plasma insulin in fasted DKO (n=4), K(n=4) and WT (n=4) and refed DKO (n=6), KO (n=4) and WT (n=6) mice. Data are means ± SD. P values were determined by unpaired two-tailed t-test. ns: not significant. f: PAS staining in DKO, KO and WT in fasted mice. Scale bar: 250 µm. Representative blot of four independent experiments. g: Glycogen content in fasted DKO (n=4), KO (n=4) and in WT (n=6) and DKO (n=5) refed mice. Data are means ± SD. P values were determined by unpaired two-tailed t-test. ns: not significant. h: Mean daylight-, night- and 24 h cumulative food intake (g/kg lean body mass/h) in DKO compared to KO mice. Data are expressed as means ± SEM of 8 animals per group. ns, not significant. i: Mean daylight-, night- and 24 h cumulative energy expenditure (kcal/kg lean body weight/h) in DKO compared to KO mice. Data are expressed as means ± SEM of 8 animals per group. ns, not significant.
Source data are provided as a Source Data file.

1 Just, P. A. *et al.* LKB1 and Notch Pathways Interact and Control Biliary Morphogenesis. *PLoS One* **10**, e0145400, doi:10.1371/journal.pone.0145400 (2015).

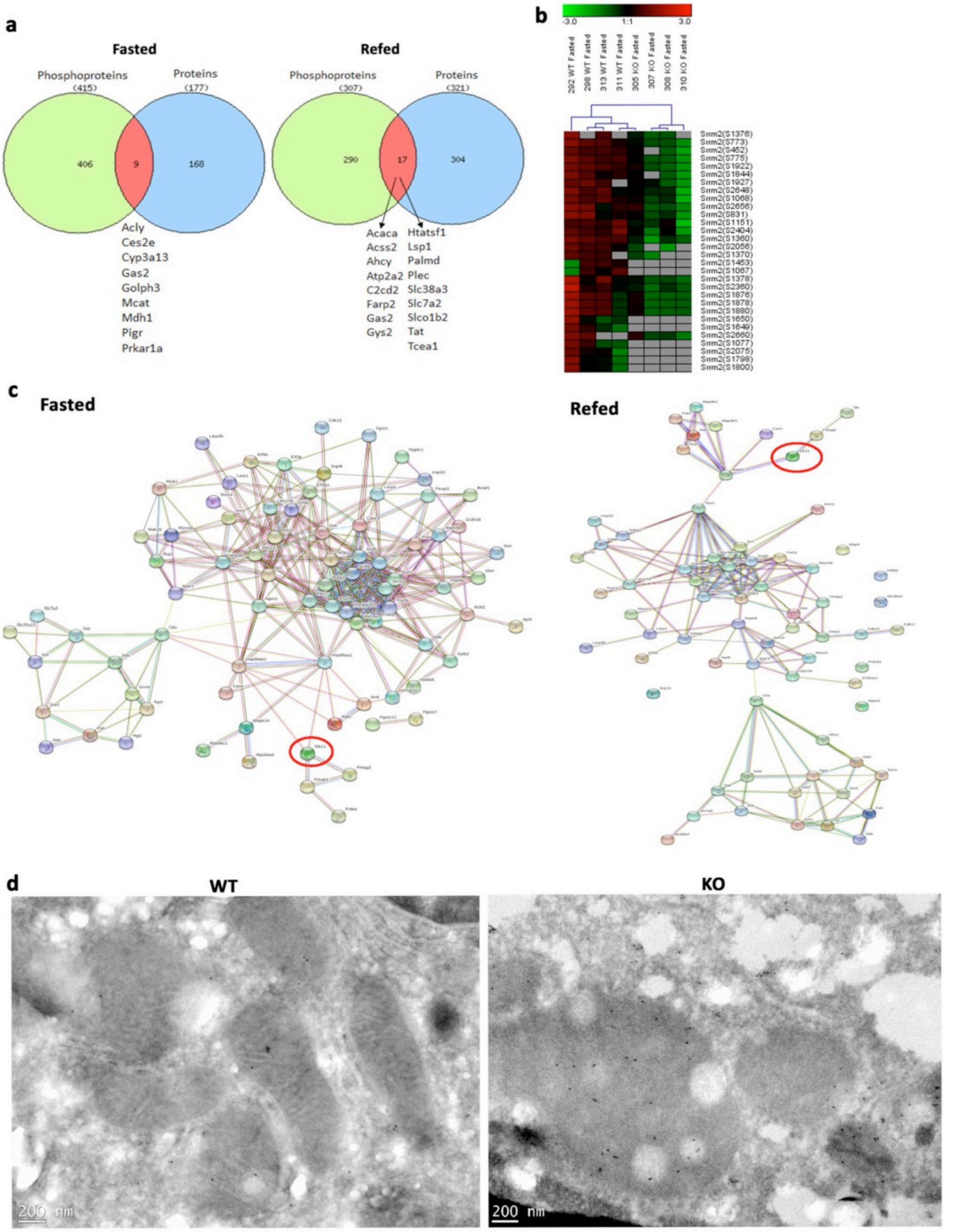

Supplementary Figure 5. Mechanisms by which Lkb1 controls the amino acid catabolism. a: Venn diagram of DE phosphoproteins (Supplementary Table 4) and DE proteins (Supplementary Table 1, Table 2). b: Heatmap of the significantly deregulated phospho-Srrm2 protein in fasted livers of Lkb1KO^{livad} and control animals. Note the numerous DE phosphopeptides for this protein. c: Interaction network between Lkb1 and phosphorylated RBP. This was done using the STRING interface, by including in each nutritional state, the DE

phosphorylated RBP, the DE kinases and phosphatases and Lkb1 (Stk11) and the metabolic targets. d: Representative gold immune-electron micrographs of Agxt in mutant and control livers. Small arrows: mitochondria. Large arrows: peroxisomes.
