# Supplementary material for: Lkb1 suppresses amino acid-driven gluconeogenesis in the liver
Source: Nat Commun. 2020 Nov 30;11:6127. doi: 10.1038/s41467-020-19490-6 (PMC7705018; doi:10.1038/s41467-020-19490-6)
Supplement: Supplementary file 3 — Description of Additional Supplementary Files [file 41467_2020_19490_MOESM3_ESM.docx]

**SUPPLEMENTARY DATA LEGENDS**

**Supplementary Data1**: Proteomic data_ Fasted WT and Lkb1KO^livad^ animals. Related to **Fig.3**. P values were determined by unpaired two-tailed t-test.

**Supplementary Data 2**: Proteomic data_ Refed WT and Lkb1KO^livad^ animals. Related to **Fig.3**. P values were determined by unpaired two-tailed t-test.

**Supplementary Data3**: Microarray for WT and Lkb1KO^livemb^ animals. Related to **Supplementary Fig.4**.

**Supplementary Data4**: Phosphoproteomic data of fasted and refed WT and Lkb1KO^livad^ animals. Related to **Fig.7**. P values were determined by unpaired two-tailed t-test.
