# Supplementary material for: Lkb1 suppresses amino acid-driven gluconeogenesis in the liver
Source: Nat Commun. 2020 Nov 30;11:6127. doi: 10.1038/s41467-020-19490-6 (PMC7705018; doi:10.1038/s41467-020-19490-6)
Supplement: Supplementary file 8 — Reporting Summary [file 41467_2020_19490_MOESM8_ESM.pdf]

nature research

Corresponding author(s): Christine Perret

Last updated by author(s): 2020/09/07

Reporting Summary

Nature Research wishes to improve the reproducibility of the work that we publish. This form provides structure for consistency and transparency in reporting. For further information on Nature Research policies, see our Editorial Policies and the Editorial Policy Checklist.

Statistics

For all statistical analyses, confirm that the following items are present in the figure legend, table legend, main text, or Methods section.

n/a	Confirmed	
☐	☒	The exact sample size (*n*) for each experimental group/condition, given as a discrete number and unit of measurement
☒	☐	A statement on whether measurements were taken from distinct samples or whether the same sample was measured repeatedly
☐	☒	The statistical test(s) used AND whether they are one- or two-sided<br>*Only common tests should be described solely by name; describe more complex techniques in the Methods section.*
☒	☐	A description of all covariates tested
☒	☐	A description of any assumptions or corrections, such as tests of normality and adjustment for multiple comparisons
☐	☒	A full description of the statistical parameters including central tendency (e.g. means) or other basic estimates (e.g. regression coefficient) AND variation (e.g. standard deviation) or associated estimates of uncertainty (e.g. confidence intervals)
☒	☐	For null hypothesis testing, the test statistic (e.g. *F*, *t*, *r*) with confidence intervals, effect sizes, degrees of freedom and *P* value noted<br>*Give P values as exact values whenever suitable.*
☒	☐	For Bayesian analysis, information on the choice of priors and Markov chain Monte Carlo settings
☒	☐	For hierarchical and complex designs, identification of the appropriate level for tests and full reporting of outcomes
☒	☐	Estimates of effect sizes (e.g. Cohen's *d*, Pearson's *r*), indicating how they were calculated

Our web collection on statistics for biologists contains articles on many of the points above.

Software and code

Policy information about availability of computer code

Data collection	Proteomic data were acquired using Maxquant version 1.6.6.0
Data analysis	Proteomic analyses were done using MaxQuant (v1.6.6.0) and Perseus softwares (v1.6.6.0)<br>The functional analyses of both proteomic and phosphoproteomic data were generated through the use of IPA (QIAGEN Inc., https://www.qiagenbioinformatics.com/products/ingenuity-pathway-analysis, v:49932394, Release Date: 2019-11-14).<br>The PPI network was done using the STRING database (https://string-db.org)<br>Heatmap were done using the genesis software (https://genome.tugraz.at)<br>Venn diagram were performed with FunRich (v3.1.3) or using the website http://www.pangloss.com/seidel/Protocols/venn.cgi.<br>Multigauge V3.0 (Fujifilm) for quantification of western blot

For manuscripts utilizing custom algorithms or software that are central to the research but not yet described in published literature, software must be made available to editors and reviewers. We strongly encourage code deposition in a community repository (e.g. GitHub). See the Nature Research guidelines for submitting code & software for further information.

Data

Policy information about availability of data

All manuscripts must include a data availability statement. This statement should provide the following information, where applicable:

- Accession codes, unique identifiers, or web links for publicly available datasets
- A list of figures that have associated raw data
- A description of any restrictions on data availability

The proteomic data were obtained using the Uniprot-Swissprot database (Uniprot, release 2015-02). Analyses of the data were done using IPA database and the STRING database.
Proteomic data that support the finding of this study have been deposited in the repository PRIDE with the accession numbers PXD013478 (https://www.ebi.ac.uk/pride/archive/PXD013478) and PXD019757 (https://www.ebi.ac.uk/pride/archive/PXD019757).
The sequencing data that support the finding of this study have been deposited in the the Gene expression Ommnibus with the accession number GSE75564 (https://www.ncbi.nlm.nih.gov/geo/query/acc.cgi?acc=GSE75564) and with the accession number GSE132536 (https://www.ncbi.nlm.nih.gov/geo/query/acc.cgi?acc=GSE132536).

Field-specific reporting

Please select the one below that is the best fit for your research. If you are not sure, read the appropriate sections before making your selection.

☒ Life sciences ☐ Behavioural & social sciences ☐ Ecological, evolutionary & environmental sciences

For a reference copy of the document with all sections, see nature.com/documents/nr-reporting-summary-flat.pdf

Life sciences study design

All studies must disclose on these points even when the disclosure is negative.

Sample size	No method was used to estimate sample size
Data exclusions	No data were excluded
Replication	All experiments were repeated and reproducibility confirmed. All experiments were done independently in most of the cases three times, unless expressed in the figure legend. Proteomic studies were done once and four biological replicates were analyzed. For sequencing studies, they were done once and three biological replicates were analyzed.
Randomization	Random
Blinding	This was not a blinded study, but mouse studies, indirect calorimetric studies, proteomic analyses and amino acid measurements were performed by different researchers. For data acquisition and data analysis, blinding was not necessary or advised to reduce any bias of the statistical analysis.

Reporting for specific materials, systems and methods

We require information from authors about some types of materials, experimental systems and methods used in many studies. Here, indicate whether each material, system or method listed is relevant to your study. If you are not sure if a list item applies to your research, read the appropriate section before selecting a response.

Materials & experimental systems

n/a	Involved in the study	
☐	☒	Antibodies
☐	☒	Eukaryotic cell lines
☒	☐	Palaeontology and archaeology
☐	☒	Animals and other organisms
☒	☐	Human research participants
☒	☐	Clinical data
☒	☐	Dual use research of concern

Methods

n/a	Involved in the study	
☒	☐	ChIP-seq
☒	☐	Flow cytometry
☒	☐	MRI-based neuroimaging

Antibodies

Antibodies used	Primary antibodies were obtained from Cell Signaling Technologies (LKB1: clone D60C5, p-Ampk (T172), #50081; Ampk,#5831; Akt, #9272; p-Akt (S473), #4060; Gys2,#3886), Abcam (Oat, ab137679; Agxt ab178708), and Santa Cruz (Gapdh FL-335;Got1,sc-515641), p-Pygl (S15) was obtained from MRC PPU reagents and services.

Validation	Commercial antibodies were validated by the suppliers, see the supplier's websites.For the MRC supplier, the exact validation process is unknown to the investigators.

Eukaryotic cell lines

Policy information about cell lines

Cell line source(s)	Primary hepatocytes extracted from liver of mice.
Authentication	N/A
Mycoplasma contamination	Not tested
Commonly misidentified lines (See ICLAC register)	N/A

Animals and other organisms

Policy information about studies involving animals; ARRIVE guidelines recommended for reporting animal research

Laboratory animals	Transgenic mice used in the study are Lkb1fl/fl,TTR-CreTam, AgxtKO, Ampkα1lox/lox, Ampkα2lox/lox, Alfp-Cre. Most of the studies were performed on male mice 15 days after injection. For the survival analysis, both males and females were monitored for up to 300 days.
Wild animals	No wild animal was used in the study
Field-collected samples	No field-collected samples was used in the study
Ethics oversight	All animal procedures were carried out according to French legal regulations and approved by ethics committee at the University Paris Descartes.

Note that full information on the approval of the study protocol must also be provided in the manuscript.
